# Melanoma Inhibitory Activity (MIA) Is Able to Induce Vitiligo-Like Depigmentation in an *in vivo* Mouse Model by Direct Injection in the Tail

**DOI:** 10.3389/fmed.2020.00430

**Published:** 2020-08-21

**Authors:** Matteo Bordignon, Roberto Luisetto, Maria Luisa Valente, Marny Fedrigo, Chiara Castellani, Annalisa Angelini, Mauro Alaibac

**Affiliations:** ^1^Unit of Dermatology, University of Padua, Padua, Italy; ^2^Department of Surgical Oncological and Gastroenterological Sciences, University of Padua, Padua, Italy; ^3^Department of Cardiac Thoracic and Vascular Sciences, University of Padua, Padua, Italy

**Keywords:** melanoma inhibitory activity, skin, vitiligo, melanocyte, autoimmunity, pathogenesis, treatment

## Abstract

In the complex pathogenesis of vitiligo, the exact mechanism of the dermatosis is still to be clarified. We previously demonstrated that a protein called melanoma inhibitory activity (MIA) is present in non-segmental vitiligo skin and seems to cause the detachment of melanocytes, consequently creating the depigmented macules. In this study, we present an animal model of vitiligo on the basis of the ability of the MIA protein to induce vitiligo-like lesions. Twenty pigmented mice were chosen for the experiments and received injections in the tail with saline (control group) or with saline + MIA protein (treated group). The control group did not show any sign of depigmentation. The treated group showed, instead, clear zones of complete depigmentation in the injected areas in each mouse, with the appearance of white patches with whitening of the hair and a clear-cut edge. Histological examination of the tail in the treated zone showed the absence of melanocytes, without the presence of any inflammatory cell or any sign of skin inflammation patterns, confirming the detachment of the melanocyte operated by the MIA protein. These data seem to confirm a possible role played by the MIA protein in the pathogenesis of vitiligo and may support the development of treatments able to inhibit its action as an alternative therapeutic strategy for this disorder.

## Background

Vitiligo is an acquired chronic pigmentation disorder of the skin that affects 0.5–2% of the population worldwide. The disease causes the formation of achromic patches on the skin surface with tendency toward symmetrical distribution and enlargement during years in its non-segmental form (1).

Despite several studies performed during years and even if the role of the immune system seems to be nowadays well-established, the exact pathogenesis of the dermatosis is still to be fully clarified.

We previously suggested that the final step of the formation of the achromic patches could be mediated by the action of a protein called melanoma inhibitory activity (MIA) (2).

MIA is a small protein firstly described as secreted from malignant melanoma cells, which is able to interact with a particular group of adhesion molecules called alpha5beta1 integrins (a5b1ints). The binding of MIA to these proteins at the cell surface is responsible for the detachment of melanocytes from extracellular matrix proteins (3). The role of MIA in normal skin was never been investigated since 2013, when we demonstrated that MIA is present also in the skin of patients affected by vitiligo, and it interacts with the same binding proteins that usually keep the melanocytes firmly attached to the basal membrane (2). These findings were consistent with the “melanocytorrhagic hypothesis” firstly described in 2003 by Gauthier et al. and recently reconsidered as a possible pathogenetic mechanism (4–6).

**Figure 1:**
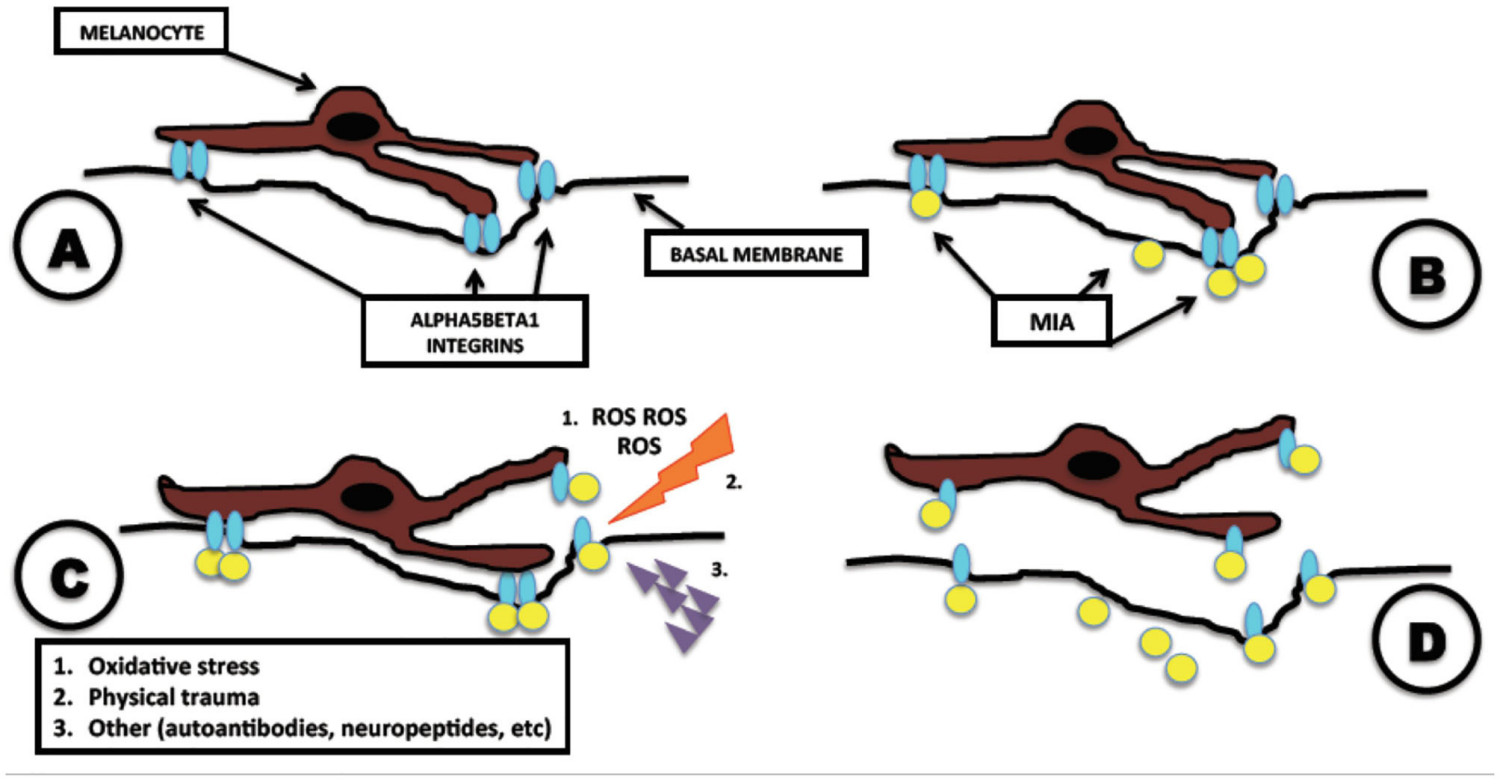
Melanoma inhibitory activity (MIA)-related pathogenesis of vitiligo. Mechanism of action of MIA in melanocytes leading to vitiligo. **(A)** Normal adhesion of melanocyte attached to the basal membrane, mediated by alpha5beta1 integrins. **(B)** Vitiligo disease: melanocyte is attacked by MIA, which binds to alpha5beta1 integrins. **(C)** After the binding of MIA with alpha5beta1 integrins, the presence of other precipitating factors as oxidative stress, physical trauma, or autoantibodies initiates the detachment of the melanocyte. **(D)** Complete detachment of melanocyte and formation of vitiligoid patches on the skin. Modified from Bordignon et al. (2).

According to this hypothesis, melanocytes, under the action of MIA, detach from the basal membrane toward the stratum corneum and exfoliate together with the surrounding keratinocytes, creating the depigmented macules (Figure 1). This process is consistent with the absence of an inflammatory response clinically evident on the vitiligo-affected skin and with the clear-cut edge of the lesions observed clinically and histologically. According to this hypothesis, melanocytes are perfectly functional and are detached singularly and silently by MIA.

In this study, we developed a mouse model of vitiligo on the basis of the action of the MIA protein, characterized by the appearance of clear zone of depigmentation determined by the detachment of melanocytes from the basal membrane.

## Materials and Methods

### Mice

Twenty pigmented mice c57bl6 type non-genetically modified were chosen for the experiments.

Furthermore, two pigmented mice c57bl6 type non-genetically modified were added after the completion of the experiments to collect images of the full sequence of depigmentation.

**Figure 2:**
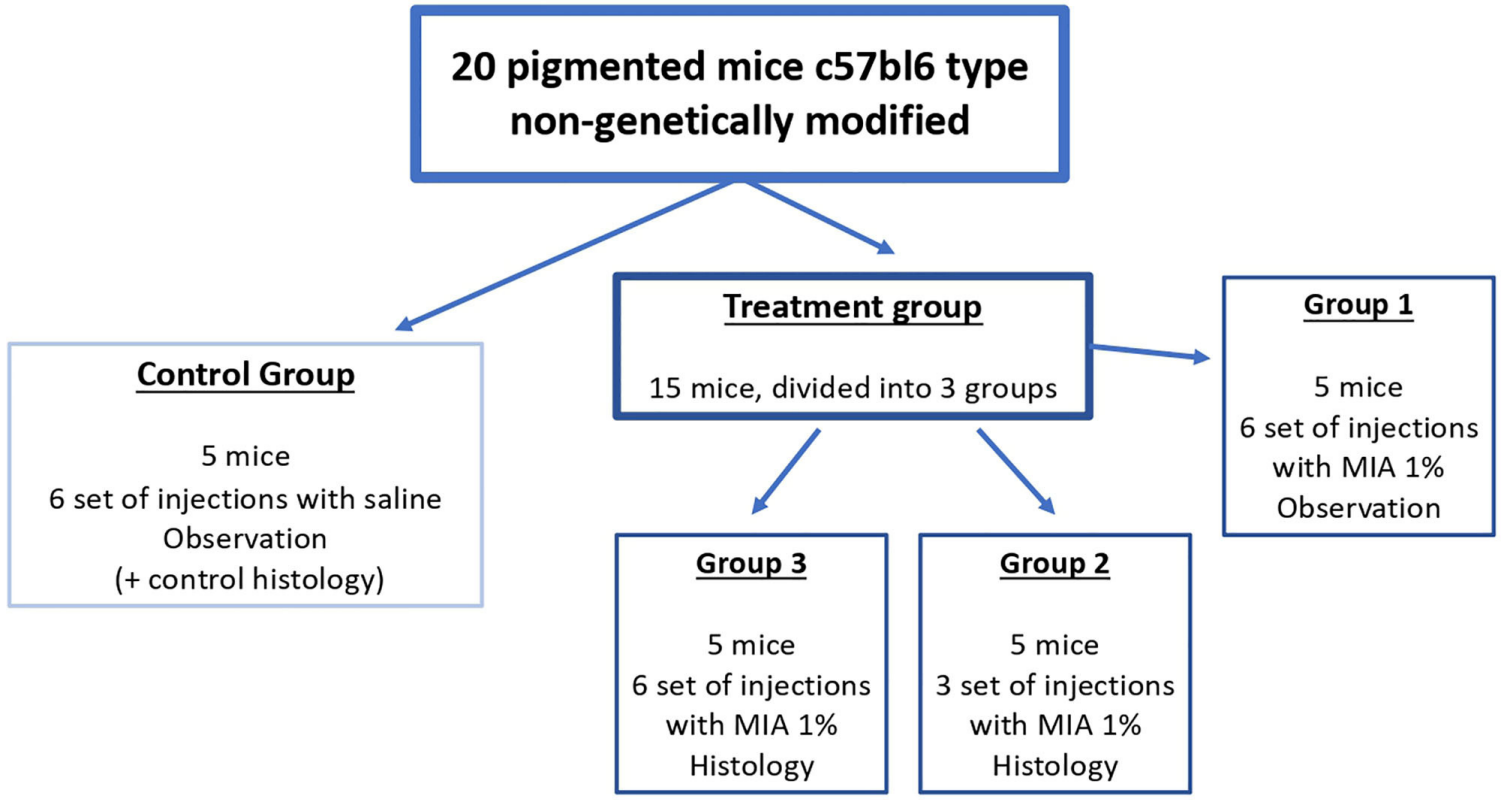
Scheme of treatment of the study. We divided the mice into four groups, one control group (treatment with saline) and three treatment groups [injection of melanoma inhibitory activity (MIA) 1% in saline]. Every injection set was performed by inoculation of 0.05 ml for each point (six injections in total, approximately every 1 cm, starting from the bottom of the tail toward the body of the mouse). We performed the injection set every 15 days (six sets in total in 3 months).

The 20 mice were randomly divided into four groups (Figure 2): control group (five mice, injection of saline solution, six sets of injections, histological observation of the tail of two mice after the final set of injections, observation of the remaining three mice), group 1 (five mice, injection of MIA 1% in saline solution, six sets of injections, observation), group 2 (five mice, injection of MIA 1% in saline solution, histological examination of the tail after three sets of injections), and group 3 (five mice, injection of MIA 1% in saline solution, histological examination of the tail after six sets of injections).

The two mice added after the completion of the experiments were treated with injections of MIA 1% in saline solution, six sets of injections.

The injections were performed with saline alone (control group) or with a solution of MIA protein (Creative BioMart, NY, USA) at 1% in saline. We inoculated 0.05 ml for each injection (six injections in total, approximately every 1 cm, starting from the bottom of the tail toward the body of the mouse). We performed the injection set every 15 days (six sets in total in 3 months). Each injection set was preceded by administration of propofol in order to obtain complete analgesia on mice.

We chose to treat only the tail of the mice because of its closest similarity to human skin as compared with other anatomic sites and owing to the technical difficulty to maintain the solution injected in contact with the epidermal compartment in other sites (e.g., ears, feet, or body hair) without being drained by the regional nodes.

All mice were maintained in pathogen-free facilities (monitored regularly) at the University of Padua, and procedures were approved by the University of Padua Institutional Animal Care and Use Committee.

### Histopathology and Immunohistochemistry

Histological examinations were performed in the tail of mice at the scheduled date for each group.

All tails were analyzed with hematoxylin and eosin (H&E) stain to evaluate the differences of normal and depigmented skin and the melanocytes from the basal membrane of epidermis. Sections obtained from each mouse tail were prepared, treated with decalcified solution, and stained for immunohistochemistry according to standard procedures.

Immunohistochemical staining with antibodies against the common leukocyte antigen (CD45 clone 2B11+PD7/6 dil 1/200, DAKO) and the pan-T antigen CD3 using 10 μg/ml rat anti-mouse CD3 monoclonal antibody (catalog # MAB4841) was performed to identify a possible skin inflammation pattern. Melan A antibody (clone SC-71566, dil 1/200, Santa Cruz Biotechnology) for the detection of premelanosomes and mature melanosomes was used to confirm presence or absence of melanocytes. Anti-mouse integrin a5b1 (polyclonal, clone orb 13515, dil 1/200; Biorbyt) and anti-MIA (clone c-10, dil:1/100; Santa Cruz Biotechnology) were evaluated to confirm the action of MIA and its regular presence in the MIA-injected mice.

## Results

All treated mice did not show any side effects locally or systemically or any signs of local inflammation or irritation due to the injection of saline or MIA solution.

**Figure 3:**
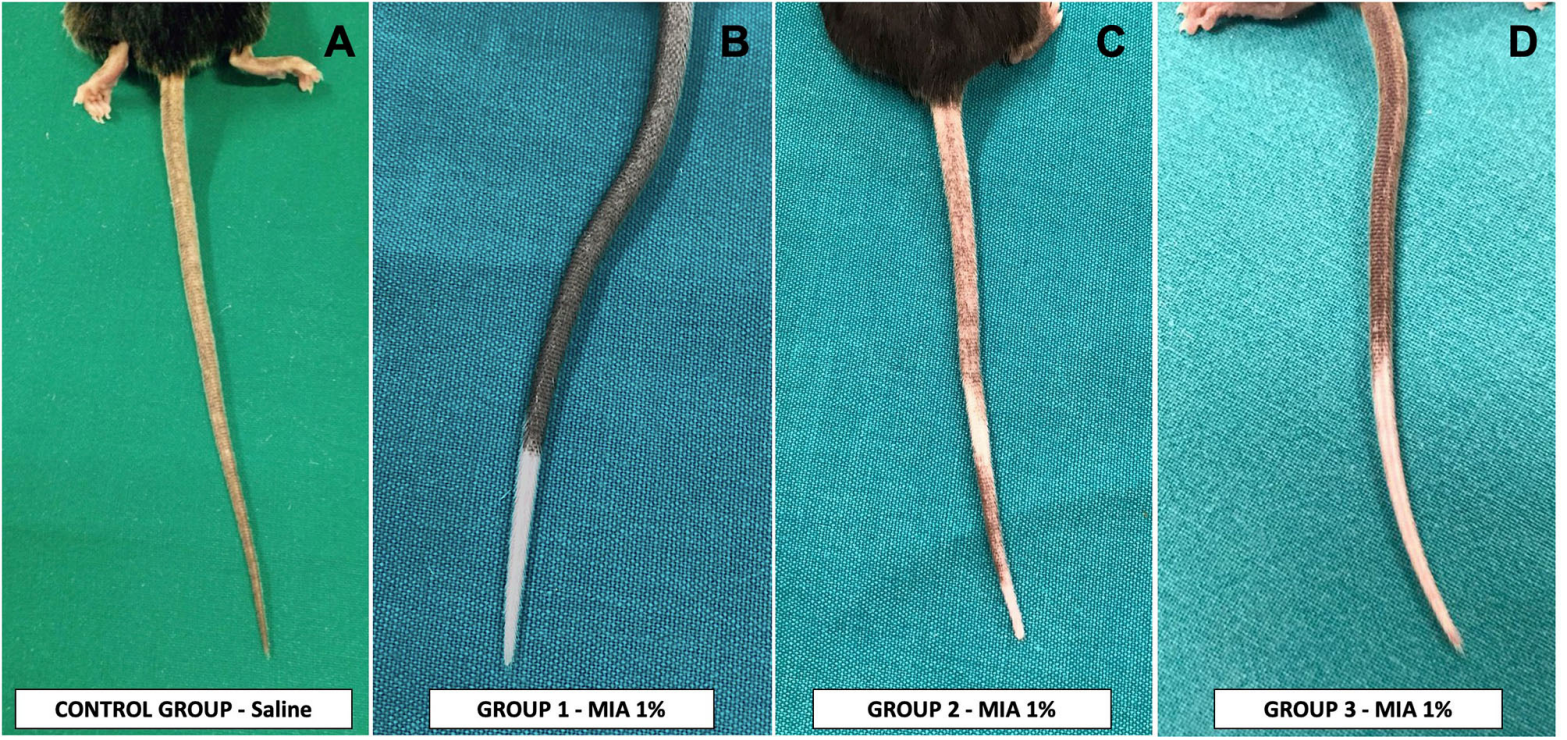
Effects of injection of melanoma inhibitory activity (MIA) or saline in the tail of mice. **(A)** Control group—injection of saline, with absence of any depigmentation and normal-appearing skin. **(B)** Group 1 and **(D)** group 3—injection of MIA 1% in saline, with the appearance of a wide and complete area of depigmentation in the treated zones, without any signs of damage or alteration of mice skin. **(C)** Group 2—injection of MIA 1% in saline, with the appearance of an incomplete area of depigmentation in the treated zones without any signs of damage or alteration of mice skin.

The control group did not show any signs of depigmentation (Figure 3A), maintaining a regularly pigmented tail immediately after all sets of injections and at the end of the period of observation (3 months more after the last set of injections).

The three treated groups showed, instead, clear zones of complete depigmentation in the injected areas in each mouse, with the appearance of white patches with whitening of the hair within the third set of injections. The depigmentation observed was mostly uniform and continuous (Figures 3B,D). Two mice of group 2 showed an alternation of depigmented and pigmented zone corresponding to the injection sites (Figure 3C). All the depigmented areas showed a clear-cut edge with respect to the pigmented areas. All the pigmented areas did not show any reduction in the pigmentation compared with their standard color. The last injection sites proximal to the body of the mice performed with MIA solution did not achieve any depigmentation in all the treated groups.

Treated group 1 showed the continuous depigmentation achieved after the sets of injections without any sign of spontaneous repigmentation after the observational period (3 months more after the last set of injections).

**Figure 4:**
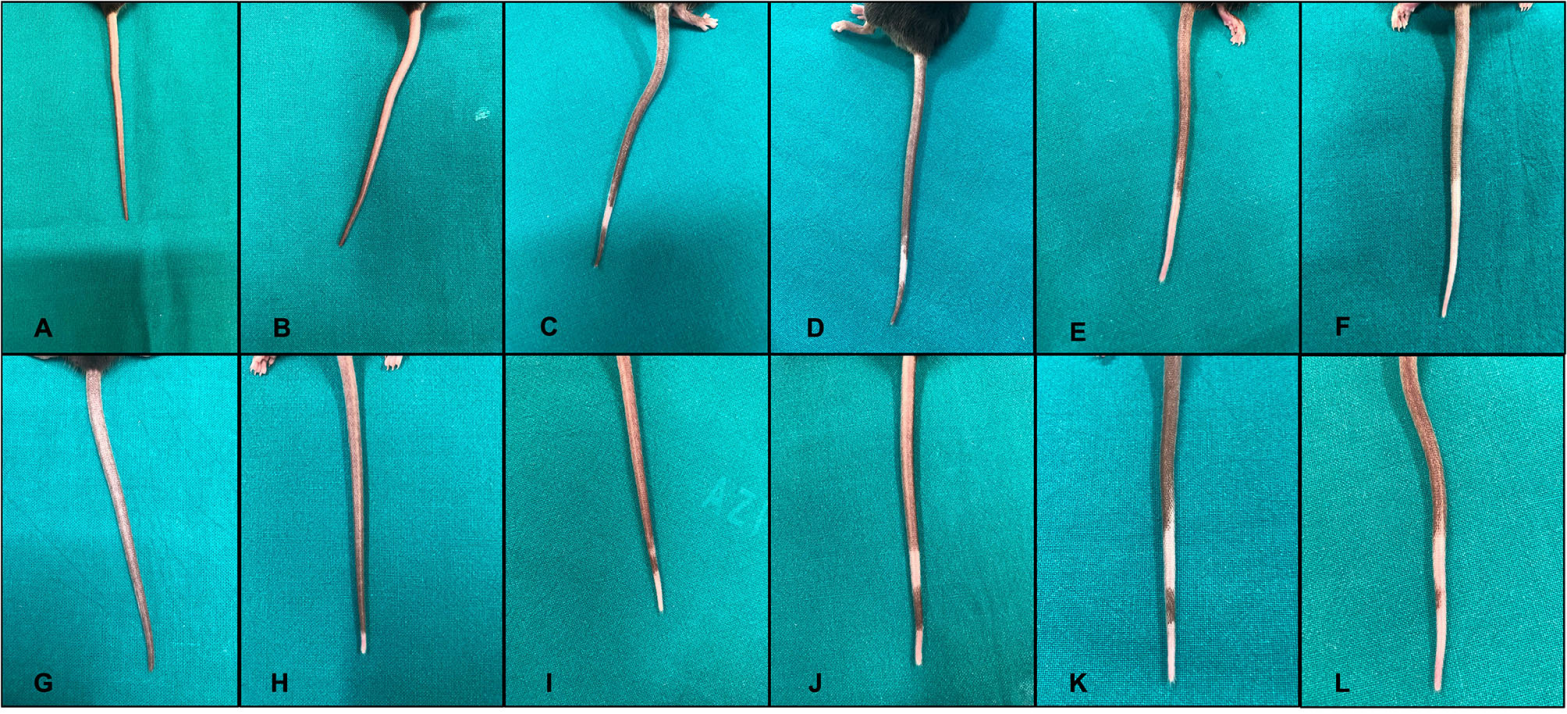
Sequence of injections of melanoma inhibitory activity (MIA) 1% solution in the tail of two different mice **(A–F)**, first mouse; **(G–L)**, second mouse. Every panel corresponds to a single set of injections. The depigmentation appears after the first second mouse; **(H)** or second first mouse; **(C)** set of injections and then spreads through the tail during the various sets of injections of the MIA protein.

To collect images of the complete sequence of depigmentation in the same mouse, we treated two additional mice with the same scheme of group 1, achieving a progressive depigmentation of the tail (Figure 4).

**Figure 5:**
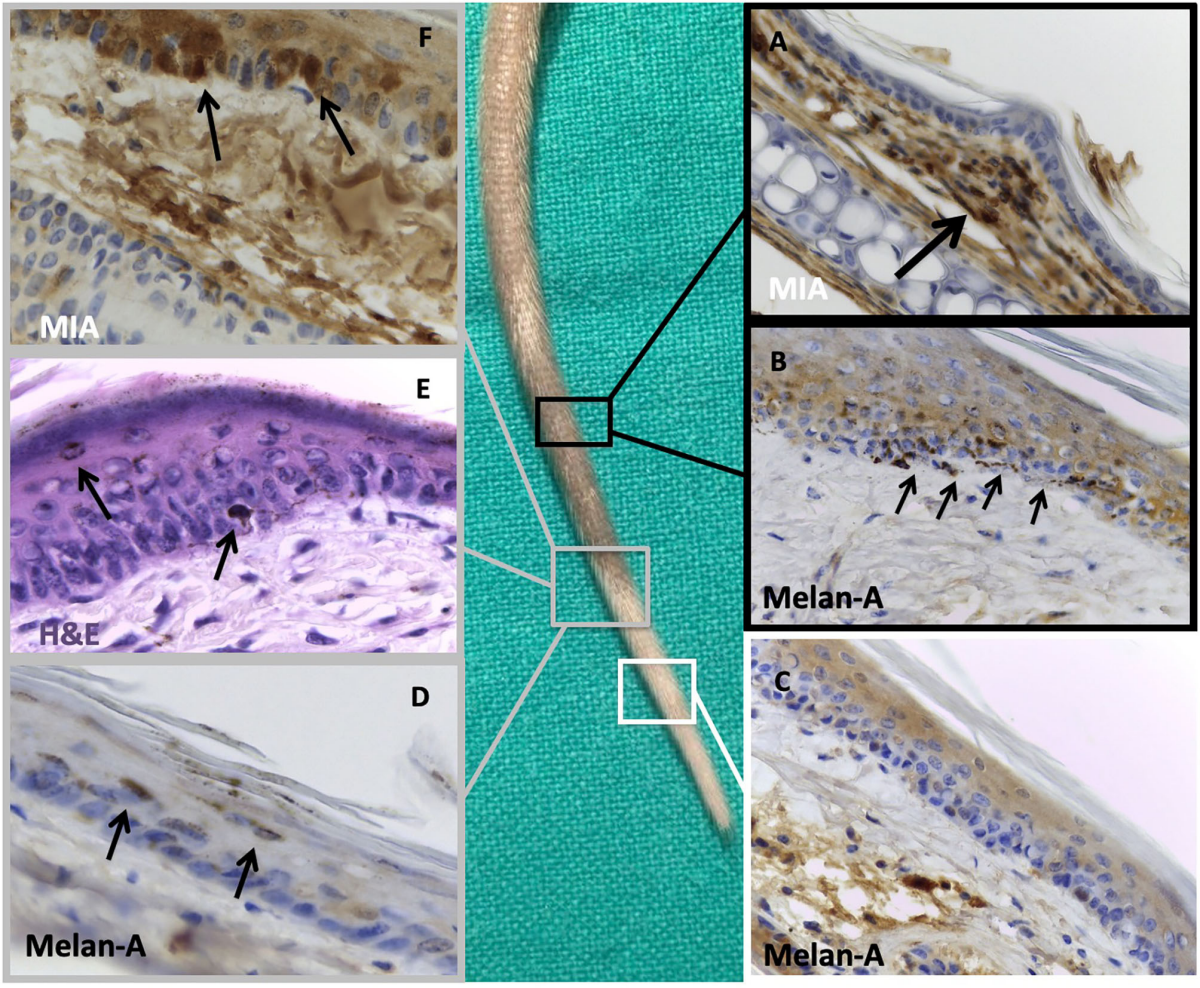
Histological examination of a treated tail focusing on pigmented and depigmented zones. Black insets: histological examination of the normo-pigmented zone where we can notice: **(A)** melanoma inhibitory activity (MIA) staining of this area, confirming the absence of the protein in the epidermal layer with finding of the protein in the dermal compartment (black arrow), probably owing to a defective injection technique, **(B)** Melan A immunostaining of normal-appearing tail skin, showing the adequate number of melanocytes in basal membrane of the epidermis (black arrows). White inset: histological examination of the completely depigmented zone where we can notice **(C)** Melan A immunostaining of depigmented tail skin with absence of melanocytes on the basal membrane of epidermis. Gray insets: histological examination of the transition zone between pigmented and depigmented areas where we can notice **(D)** Melan A staining and **(E)** hematoxylin and eosin staining of the transition zone between pigmented and depigmented tail skin with presence of some detached melanocytes toward the stratum corneum (black arrows); **(F)** MIA staining of the tail confirming its regular presence in the epidermal compartment (black arrows). Original magnification, 40 ×.

Histological examination of the tail showed clear differences among the control and the treated groups regarding the presence of melanocytes, as demonstrated by Melan A staining. The control group (and the non-depigmented zone of the injected mice) showed a normal pattern of melanocytes distribution (Figure 5B), whereas the treated groups showed the absence of melanocytes corresponding to the clinical depigmentation (Figure 5C). In the transition zone of the treated groups, some detaching melanocytes could be detected at various levels through the epidermis of the mice (Figures 5D,E).

Notably, “attached melanocytes” are defined as melanocytes completely adherent to the basal membrane in the epidermal layer, whereas “detached melanocytes” are defined as melanocytes not adherent to the basal membrane and could be found in the epidermal layer at different levels.

The staining for integrin a5b1 was positive for the presence of the adhesion molecule in the melanocytes, particularly in the exfoliating ones.

The depigmented sections were strongly positive for the presence of MIA in the transition zone (Figure 5F). Interestingly, the part of the tails that remained pigmented also in the mice treated with the 1% MIA solution showed no presence of MIA (Figure 5A) in the epidermal layer.

In all the specimens analyzed, we found no presence of inflammatory cells or histological signs of skin inflammation.

## Discussion

The complex pathogenesis of vitiligo is still to be fully clarified. The role of the immune system is well-established, but some mechanisms of the disease need to be fully elucidated.

An alternative hypothesis to explain vitiligo development was the melanocytorrhagic hypothesis, which considered vitiligo as a disease caused by chronic detachment and transepidermal loss of melanocytes (4–6). This detachment would be the silent manner by which the vitiliginous skin eliminates its pigment away, as we commonly observe in our vitiligo-affected patients. The cause of detachment could be a defective adhesion system connecting melanocytes to the basal membrane as recently proposed (6–8) or the presence of another factor able to break these connections. In this investigation, we demonstrated that MIA protein could be a possible molecule able to operate the detachment (2). We previously demonstrated that this protein, present and operative in the skin of vitiligo-affected patients, is able to interact directly with the adhesion complex of melanocytes (notably, with a5b1ints) and to interrupt these connections, leading to the cell exfoliation with the surrounding keratinocytes. According to both these observations and previously published data about the activity of the protein (9), MIA is produced by the melanocytes themselves, and it is assembled in a tetrameric form against integrins of vitiligo-prone melanocytes, weakening their anchorage to the basal membrane and favoring their detachment and exfoliation as we clearly showed in a patient with an active form of vitiligo (2). In order to investigate the melanocytorrhagic hypothesis, we established a mouse model of vitiligo on the basis of the sole action of the MIA protein. The appearance of a clear and complete depigmentation zone in the mice tail just after the first sets of injections confirmed that MIA was able to directly detach melanocytes. The depigmentation created by the MIA protein was fully compatible clinically, histologically, and immunophenotypically with that observed in human vitiligo: clear-cut transition zone among normal and white skin, involvement of annexes, absence of any inflammatory sign, and perfect normal appearance of the affected skin (see Figures 3, 4).

The histological examination performed on the tail confirmed that the depigmentation created was due to the absence of melanocytes, as already demonstrated in human vitiligo. Particularly, we were able to detect some exfoliating melanocytes in the transition zone, confirming the melanocytorrhagic effects of MIA as we observed in vitiligo-affected patients. Moreover, we found that the part of the tail still pigmented after the injection of MIA-containing solution did not present a histological positivity for the protein in the epidermis. The lack of protein detection could be due to a defective injection technique in that side (with the insertion of the solution in the deeper dermis and not in the epidermal layer or close to it, with dispersion of the protein) (see Figure 5A) or more likely to the action of the local nodes of the mice able to eliminate every external protein introduced in the animals. Because the MIA protein acts as a tetramer (9), the entire process of assembling, docking to the integrins, and cleaving required a certain period of time of unperturbed action. The draining of the nodes with sequestration of the MIA protein made this action more difficult as long as we performed the injection closer to these nodes. This observation may also indicate that the action of cleavage of MIA protein against the adhesion molecules was very efficacious as only the absence of the protein left these molecules unperturbed.

Our negative findings for the presence of immune cells in this mechanism may indicate that the final step of vitiligo development could not require an autoimmune stimulation. The absence of an involvement of the immune system in our model was confirmed by the histological analysis of the treated group 2, where we sacrificed the mice at the very beginning of the depigmentation (after the third set of injections) to verify that also at this stage the immune system action was absent and the only mechanism operative was the action of the MIA protein. This observation also confirmed our previous findings in human vitiligo, where all skin samples positive for the MIA protein were negative for the presence of immune-related cells.

Based on our previous demonstration of the presence of MIA protein in the skin of patients affected by vitiligo and on the ability to create a vitiligo-like depigmentation in mice, this molecule could probably be considered as a key part of the very complex puzzle of vitiligo pathogenesis.

If we can consider the MIA protein as the real and direct “killer” of melanocytes, it is not clear the reasons why it is produced by melanocytes themselves. About the immunological relevance of this mechanism, surely a lot of actors could be operative in both dermal and epidermal compartments, involving certainly the immune system cells, oxidative stress, and mechanism of senescence of the melanocytes.

Considering all the previous data about the action of the immune system in the skin and the recent findings about the action of the MIA protein, it is possible to connect these various aspects in a unitary model. It is well-known that key molecules in the autoimmune response are interferons (IFNs) (10). IFNs may have a key role also in vitiligo, as the treatment of different diseases using IFNs may be associated with the onset of vitiligo, notably metastatic melanoma (11), hepatitis C infections (12), or during the treatment of skin tumors with imiquimod, an IFN gamma (IFNγ)-releasing agent (13).

IFNγ was described as one of the main molecules able to drive the melanocytes toward a senescent phenotype (14), which is one of the predominant features when analyzing vitiligo-prone melanocytes (15). Because it has been recently reported that the production of the MIA protein is associated with the senescent phenotype of melanocytes (16), it is plausible that genetically prone melanocytes in predisposed individuals (10, 17) would develop a senescent phenotype mediated by IFN. These melanocytes, probably under the action of other stimuli like local friction (18) or oxidative stress (19), would then start to produce the MIA protein, which in turn could be responsible for the detachment of melanocytes from the basal membrane, developing the depigmented patches of vitiligo on the skin surface.

More studies are required to confirm this hybrid mechanism with particular regard to the dynamic interplay involving the immune system, genetic predisposition, and the MIA action. Furthermore, reliable quantitative methods of immunohistochemical identification need to be developed in future studies in order to determine the proportion of detached melanocyte in the epidermal layer induced by MIA and expression of the MIA protein. These data will be necessary to completely support our conclusions.

## Conclusions

MIA protein causes complete vitiligo-like depigmentation by direct injection in the tail of mice, operating a melanocythorragic effect on the melanocytes. These data together with the previous observation of the presence of MIA in the skin of vitiligo-affected patients suggest a role played by this protein in the disease and support the development of treatments able to inhibit its action as an alternative therapeutic strategy for this disorder.

## Data Availability Statement

The datasets generated for this study are available on request to the corresponding author.

## Ethics Statement

The animal study was reviewed and approved by Ethical committee University Hospital of Padova.

## Author Contributions

All authors listed have made a substantial, direct and intellectual contribution to the work, and approved it for publication.

## Conflict of Interest

The authors declare that the research was conducted in the absence of any commercial or financial relationships that could be construed as a potential conflict of interest.
